# Toll-Like Receptor 2 or Toll-Like Receptor 4 Deficiency Does Not Modify Lupus in MRLlpr Mice

**DOI:** 10.1371/journal.pone.0074112

**Published:** 2013-09-24

**Authors:** Simon J. Freeley, Angela Giorgini, Calogero Tulone, Reena J. Popat, Catherine Horsfield, Michael G. Robson

**Affiliations:** 1 MRC Centre for Transplantation King’s College London, London, United Kingdom; 2 Department of Histopathology, Guy’s and St Thomas’ NHS Foundation Trust, London, United Kingdom; Beth Israel Deaconess Medical Center, United States of America

## Abstract

Systemic lupus erythematosus is an autoimmune disease with a high morbidity and nephritis is a common manifestation. Previous studies in murine lupus models have suggest a role for Toll-like receptor 2 and 4. We examined the role of these molecules in MRL lpr mice which is one of the most established and robust murine models. We compared disease parameters in Toll-like receptor 2 or Toll-like receptor 4 deficient mice with their littermate controls. We found no difference in the severity of glomerulonephritis as assessed by histology, serum creatinine and albuminuria when Toll-like receptor 2 or Toll-like receptor 4 deficient MRLlpr mice were compared with Toll-like receptor sufficient controls. We also found similar levels of anti-dsDNA and anti-ssDNA antibodies. These results show that Toll-like receptor 2 and Toll-like receptor 4 do not play a significant role in MRLlpr mice, and therefore they may not be important in human lupus.

## Introduction

Systemic lupus erythematosus (SLE) is a systemic autoimmune disease characterised by involvement of many organs including skin, joints, kidneys and brain. Autoimmunity to nucleic acid associated antigens, including dsDNA, histones, and RNA associated proteins leads to immune complex formation and tissue damage. Glomerulonephritis is a particularly serious clinical manifestation and can lead to irreversible renal failure. Toll-like receptors (TLRs) are a family of structurally related molecules that recognise pathogen-associated molecular patterns. They can be broadly divided into cell-surface receptors such as TLR2 and TLR4 recognising bacterial products, and intracellular receptors such as TLR3, TLR7, TLR8 and TLR9 recognising nucleic acids. They have multiple effects on leukocytes and stromal cells, and influence both innate and adaptive immunity. Previous work has shown that endotoxin, now known to be a TLR4 agonist, can exacerbate disease in a variety of strains of lupus-prone mice [1]. More recently it has been shown that exogenously administered agonists of TLR3, TLR7 and TLR9 can exacerbate murine lupus [1], [2], [3], [4]. In addition several studies using the nephrotoxic nephritis model have shown a role for TLR2 and TLR4 through a number of mechanisms [5], [6], [7], [8]. Together these data support the idea that TLRs play a role in the exacerbation of lupus by bacterial and viral infection, as can be observed clinically.

In addition the potential roles of TLRs in the absence of exogenous ligand administration has been explored. Published work suggests several mechanisms by which TLR7 and TLR9 could facilitate the loss of tolerance to nucleic acid-associated antigens through B cells and dendritic cells [9], [10], [11], [12]. The role of TLR9 and TLR7 has been tested in vivo in murine models of SLE. Initial results on the role of TLR9 in the MRL/lpr mouse were conflicting, but this appears to been resolved with the conclusion that there are less autoantibodies, but worse nephritis in TLR9 deficient lupus-prone mice [13]. TLR7 deficient lupus prone mice on the other hand show less autoantibodies and less severe disease in both the MRL/lpr model and a transgenic lupus model [13], [14]. Recent work has suggested that TLR2 and TLR4 may also play a role in murine lupus in the absence of exogenous ligand administration [15]. C57BL/6 (lpr/lpr) mice deficient in TLR2 or TLR4 were studied and found to have reduced anti-dsDNA antibodies and glomerular IgG deposits, though anti-nucleosome antibodies were not affected. Pristane-induced lupus was studied in C57BL/6 TLR4 deficient mice and both autoantibodies and nephritis was found to be ameliorated compared to controls [16].

Nephritis is relatively mild in both C57BL/6-lpr mice and in pristine-induced lupus. In contrast MRL/Mp-*Tnfrsf6^lpr/lpr^* mice (abbreviated to MRLlpr) mice are one of the most robust and frequently used models of murine lupus, and mice develop severe nephritis by 18 weeks of age. There have been no publications addressing the role of TLR2 or TLR4 in this model. We therefore decided to examine if the findings suggesting a role for TLR2 and TLR4 in the milder lupus models would be confirmed in MRLlpr mice.

## Materials and Methods

### Ethics Statement

All animal experiments were performed according to Institutional and Home Office regulations (PPL 70/6617). All efforts were made to minimize suffering.

### Mice

TLR2 and TLR4 deficient mice originally obtained from S Akira and backcrossed to C57BL/6 were mated with MRL/Mp-*Tnfrsf6^lpr/lpr^* mice (abbreviated to MRL-lpr) purchased from Harlan UK. TLR deficient mice were each backcrossed 7 generations with MRLlpr mice with presence of the TLR2 or TLR4 knockout locus confirmed by PCR. After 7 generations, we confirmed with PCR that all mice were homozygous for the *Tnfrsf6^lpr/lpr^* mutation. Since MRL mice are MHC haplotpye H2^k^ and C57BL/6 mice are H2^b^, we confirmed that, after 7 generations of backcrossing, all mice were homozygous for H2^k^ using PCR and restriction enzyme digestion according to a published method [17]. Backcrossed mice heterozygous for TLR2 were interbred to give mice that were homozygous for TLR2 deficiency (denoted MRLlprTLR2−/−) or TLR2 sufficient littermate controls (denoted MRLlprTLR2+/+). PCR primer pairs were as follows. TLR2 deficient 5′-GTT TAG TGC CTG TAT CCA GTC AGT GCG-3′ and 5′-TTG GAT AAG TCT GAT AGC CTT GCC TCC-3′. TLR2 wildtype 5′-ATC GCC TTC TAT CGC CTT CTT GAC GAG-3′ and 5′-TTG GAT AAG TCT GAT AGC CTT GCC TCC-3′. Mice heterozygous for TLR2 were not studied. MRLlprTLR4−/− mice and MRLlprTLR4+/+ littermate controls were generated in a similar fashion. PCR primer pairs were as follows. TLR4 deficient 5′-CGT GTA AAC CAG CCA GGT TTT GAA GGC-3′ and 5′-TGT TGC CCT TCA GTC ACA GAG ACT CTG-3′. TLR4 wildtype 5′-TGT TGG GTC GTT TGT TCG GAT CCG TCG-3′ and 5′-TGT TGC CCT TCA GTC ACA GAG ACT CTG-3′.

Experimental cohorts were established and allowed to age to 18 weeks, with surviving mice killed at 18 weeks. Therefore all mice used in the experimental cohorts underwent PCR analysis for both TLR2 or TLR4 deficiency and the corresponding wildtype loci. Therefore there can be no doubt that all mice were correctly assigned to TLR2−/−, TLR4−/− or control groups. In addition, all mice were H2^k^ and were homozygous for the *Tnfrsf6^lpr/lpr^* mutation as indicated above.

### Histology

Renal tissue was fixed in Bouin’s solution, embedded in paraffin, stained with Period acid-Schiff, and evaluated by an expert renal pathologist (CH). Glomerulonephritis was graded 0–3 according to the following scheme: grade 0, normal; grade 1, endocapillary proliferation (>50% of the glomerulus) in 25–50% of glomeruli; grade 2, endocapillary proliferation (>50% of the glomerulus) in 50%–75% of glomeruli; grade 3, endocapillary proliferation (>50% of the glomerulus) in >75% of the glomeruli or crescents in >25% of glomeruli. A minimum of 50 glomeruli per sample were assessed for the analysis. Kidney was frozen in isopentane, and cryostat sections were stained with FITC conjugated goat anti-mouse IgG (Jackson’s Immunoresearch), or with monoclonal rat anti-mouse C3 (Cedar Lane) followed by DyLight488 mouse anti-rat IgG (Jackson’s Immunoresearch). To quantify IgG and C3 deposition, a minimum of 20 glomeruli per section were photographed at×200 magnification with an Olympus BX51 fluorescent microscope. Glomeruli were manually drawn around using ImageJ software (National Institute of Health), and the mean fluorescence intensity within the defined area measured to give a quantitative value. This process was performed without knowledge of sample identity.

### Serology and Biochemistry

Mice were bled at 14 weeks, and blood was also taken at 18 weeks from surviving mice. Spot urines were taken from mice at 14 and 18 weeks. Serum and urine creatinine was measured by electrospray mass spectroscopy in a clinical biochemistry laboratory. Urinary albumin was measured using an ELISA from Bethyl laboratories (Montogomery, Texas, USA) according to the manufacturer’s instructions. Anti-dsDNA antibodies were measured by ELISA. Nunc Maxisorb ELISA plates (Fischer Scientific, Leicester, UK) were coated with poly-L-Lysine, then with 5 µg/ml of calf thymus DNA (Sigma, Poole, UK) in TE buffer. Plates were treated with S1 nuclease (Sigma) in 30 mM sodium acetate, pH 4.6, 50 mM NaCl, 1 mM ZnCl2, in order to remove single stranded DNA. For the anti-ssDNA ELISA, wells were coated with 5 µg/ml of calf thymus DNA which had been heated at 100’C for 10 minutes. For the antidsDNA and anti-ssDNA ELISAs, samples were applied at 1 in 2000 and 1 in 800 dilutions respectively, followed by an alkaline phosphatase conjugated goat anti-mouse Ig detection antibody (Southern Biotechnology). A standard curve was generated from a reference serum and levels in samples were expressed in arbitrary units relative to this standard. Total serum IgG subclasses and IgM were assessed by capture ELISA. The capture antibody was 5 µg/ml of goat anti-mouse immunogloblin (Southern Biotech), and serum was applied at an appropriate dilution (1 in 128,000 for IgG subclasses and 1 in 4000 for IgM). Alkaline phosphatase conjugated goat antibodies (Southern Biotechnology) was used for detection, and were specific for IgG1, IgG2a, IgG2b, IgG3 or IgM. For each ELISA a standard curve was generated from a reference serum and levels in samples were expressed in arbitrary units relative to this standard. *Crithidia Lucillae* titre was measured using commercial slides (Binding Site, Birmingham, UK) and the lowest titre at which fluorescence was positive by a blinded observer assessed with a BX51 fluorescent microscope (Olympus, Southend-on-sea, UK).

### Statistics

Data were analysed with Graphpad prism (San diego, CA) software using a Mann-Whitney U for Crithidia titres and histology scores. A Student’s t-test was used for other data, after a logarithmic transformation of the data if appropriate. Survival curves were analysed with a logrank test.

## Results

### Nephritis is not Modified by TLR2 or TLR4 Deficiency

**Figure 1:**
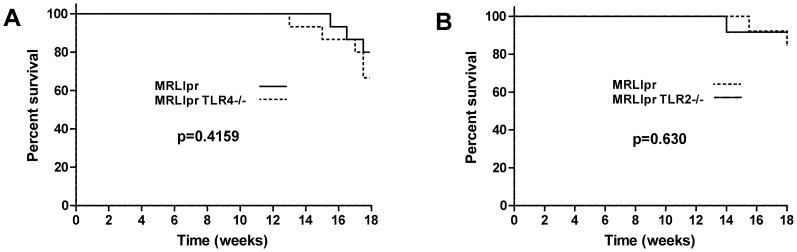
The effect of TLR2 or TLR4 deficiency on survival. **A**. Survival data for MRLlpr mice and TLR4 deficient littermate controls. **B**. Survival data for MRLlpr mice and TLR2 deficient littermate controls. There were no significant differences in either case.

**Figure 2:**
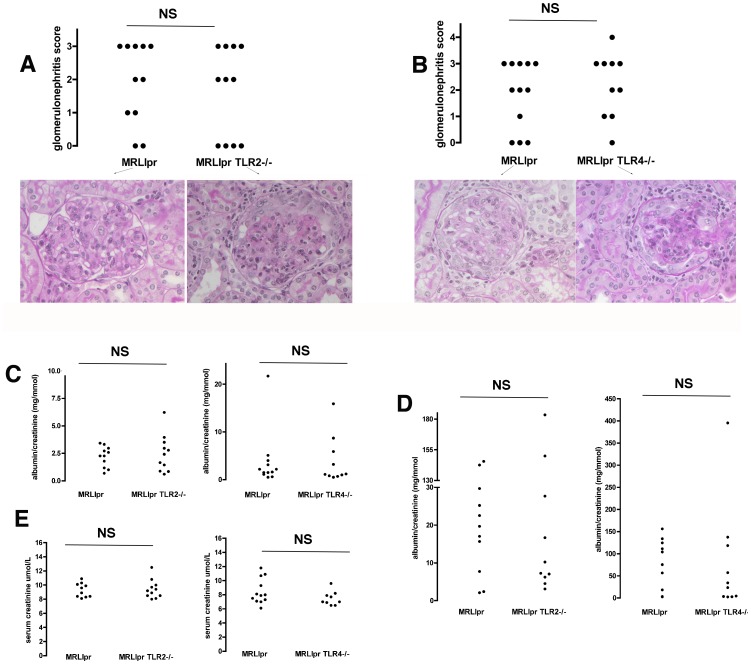
Histological and functional assessment of glomerulonephritis. **A**. Histology data showing severity of glomerulonephritis for MRLlpr mice and TLR2 deficient littermate controls at 18 weeks with representative histology, showing varying degrees of proliferative nephritis, on PAS stained sections shown below. **B**. Histology data showing severity of glomerulonephritis for MRLlpr mice and TLR4 deficient litter mate controls at 18 weeks, with representative histology on PAS stained sections shown below. There were no significant differences for either analysis. Each symbol is an individual mouse. **C**. Urine albumin creatinine ratio at 14 weeks for MRLlpr mice and TLR2 deficient littermate controls, and for MRLlpr mice and TLR4 deficient littermate controls. **D**. Urine albumin creatinine 18 weeks for these same groups. (Note one data point is missing in the TLR4 deficient group at week 18 as no urine was obtained). **E**. Serum creatinine at 18 weeks in MRLlpr mice and TLR2 deficient littermate controls and for MRLlpr mice and TLR4 deficient littermate controls. Each symbol is an individual mouse. NS = not significant.

**Figure 3:**
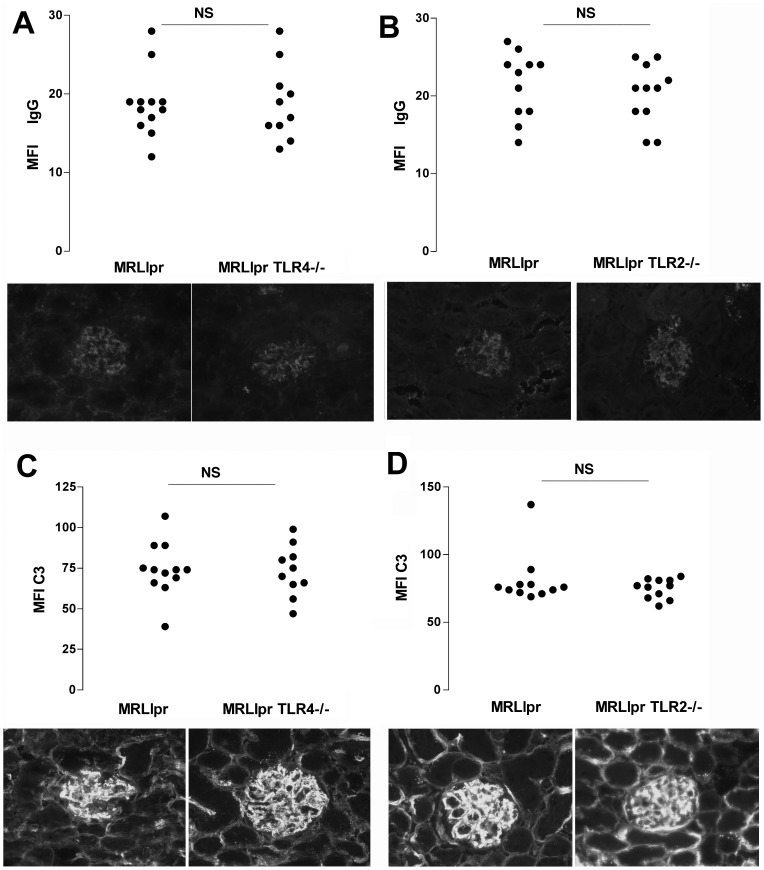
Glomerular deposition of IgG and C3 was assessed as described under methods. **A–B** shows data for IgG deposition in the two experiments with TLR deficient mice compared with littermate controls, and **C–D** shows data for C3 deposition. Each symbol is an individual mouse. NS =  not significant. A photomicrograph showing representative immunofluorescence staining is shown underneath the corresponding group of mice in panels **A–D**.

We first assessed the severity of kidney disease in cohorts of mice at 18 weeks. In the first experiment, 13 MRLlpr and 11 TLR2 deficient MRLlpr sex-matched littermate controls were compared. All mice survived to 14 weeks, though by 18 weeks two TLR2 sufficient mice had died, leaving 11 in each group for analysis. In the second experiment, 15 MRLlpr and 15 TLR4 deficient MRLlpr sex-matched littermate controls were compared. One TLR4 deficient mouse died at 13 weeks, and by 18 weeks 4 more TLR4 deficient mice and 3 TLR4 sufficient mice had died (not significant), leaving 12 TLR4 sufficient and 10 TLR4 deficient MRLlpr mice for analysis respectively. There were no significant differences in survival curves for either experiment as shown in figure 1. Glomerulonephritis was histologically graded, and there was no significant difference between MRLlpr and their TLR2 deficient MRLlpr littermate controls. In addition there was no significant difference in histology between MRLlpr and their TLR4 deficient MRLlpr littermate controls. Figure 2A–B shows histology data and representative histology. In order to assess the functional severity of disease, we measured urinary albumin creatinine ratio at both week 14 and week 18, and serum creatinine at week 18. Again no significant differences were detected between groups. These functional data are shown in figure 2C–E. We also analysed glomerular deposition of IgG and C3 in all mice from each experiment and no differences were found, as shown in figure 3.

### Autoimmunity is not Modified by TLR2 or TLR4 Deficiency

**Figure 4:**
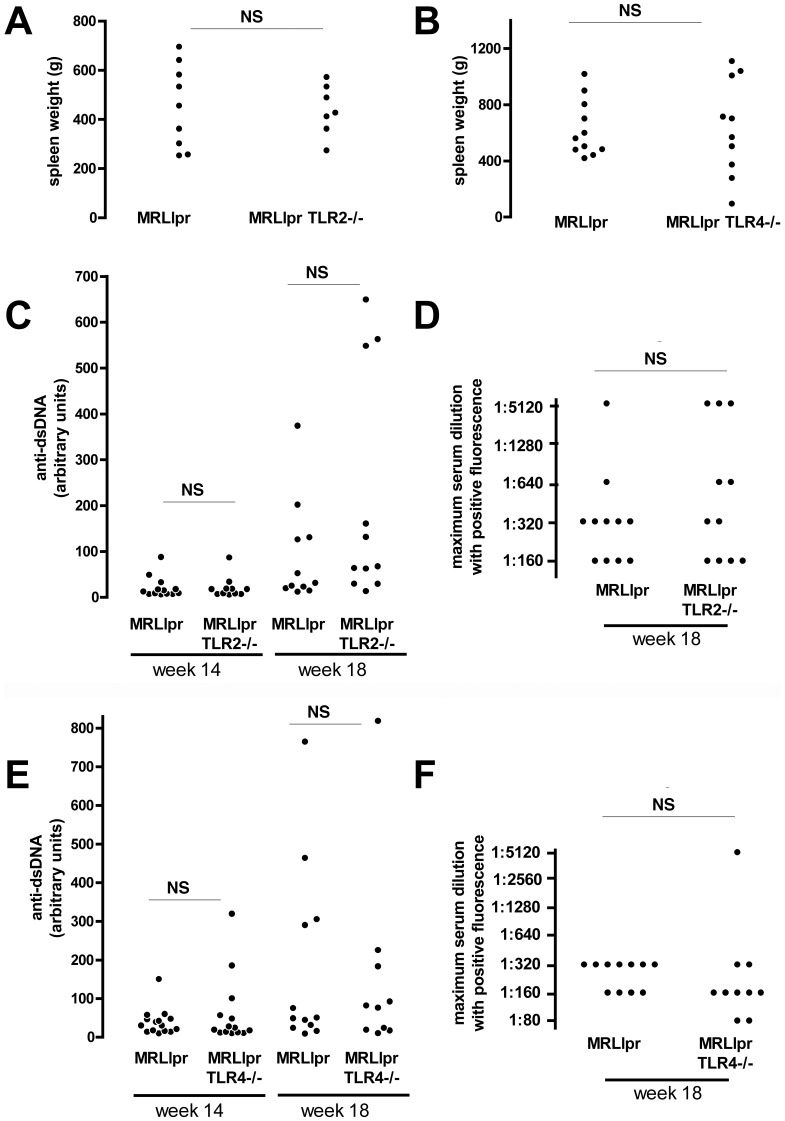
Assessment of autoimmunity. **A–B**. Spleen weights for MRLlpr mice and their TLR2 or TLR4 deficient littermate controls at 18 weeks (Note six data points were lost in panel B to a technical error). **C**. Anti-dsDNA antibodies measured by ELISA (left) at 14 and 18 weeks in MRLlpr mice and TLR2 deficient littermate controls. **D**. Anti-dsDNA antibodies were also measured by *Crithidia Lucillae* fluorescence at 18 weeks in MRLlpr mice and TLR2 deficient littermate controls. **E**. Anti-dsDNA antibodies measured by ELISA (left) at 14 and 18 weeks in MRLlpr mice and TLR4 deficient littermate controls. **F**. Anti-dsDNA antibodies were also measured by *Crithidia Lucillae* fluorescence at 18 weeks in MRLlpr mice and TLR4 deficient littermate controls. Each symbol is an individual mouse. NS = not significant.

**Figure 5:**
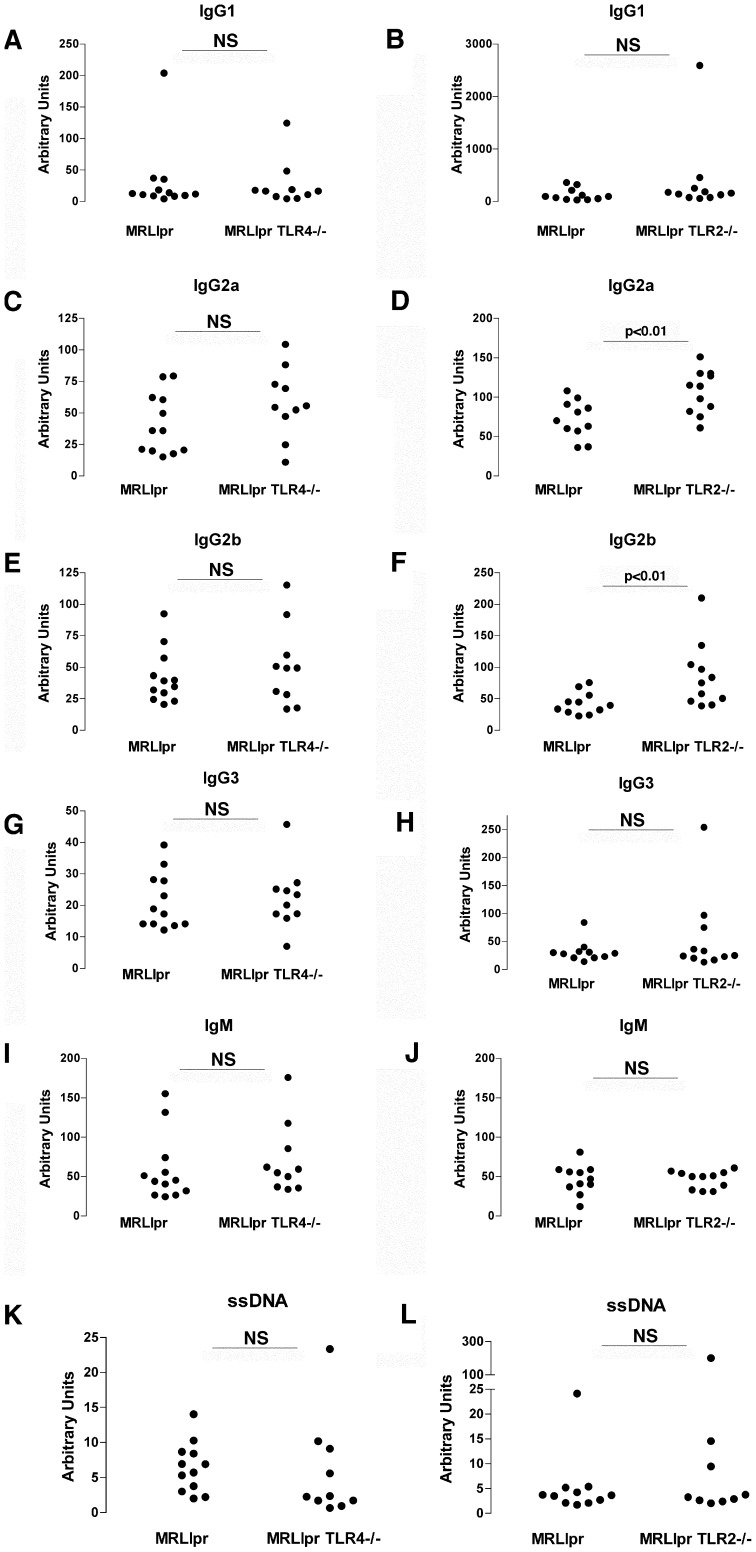
Assessment of immunoglobulin subclasses and anti-ssDNA antibodies. Relative levels of total IgG1 (**A–B**), IgG2a (**C–D**) IgG2b (**E–F**), IgG3 (**G–H**) and IgM (**I–J**) were compared in the serum of TLR deficient mice and littermate controls for each experiment. We also assessed serum levels of antibodies to ssDNA (**K–L**) in each experiment. NS = not significant. Each symbol is an individual mouse.

Although we had found no evidence that kidney disease was modified by the absence of TLR2 or TLR4, it remained possible that there was modulation of autoimmunity in TLR2 or TLR4 deficient MRLlpr mice. However there was no difference in spleen weights between MRLlpr and TLR4 deficient MRLlpr littermate controls and also no difference when MRLlpr and TLR2 deficient MRLlpr littermate controls were compared, as shown in Figure 3A. We also measured anti-dsDNA antibodies, in all mice at both 14 and 18 weeks using an ELISA-based method. We confirmed the week 18 data with a semi-quantitiative *Crithidia Lucillae* assay, and all mice had anti-dsDNA antibodies detected by both assays. There were no differences between TLR2 deficient MRLlpr TLR2−/− mice and littermate controls (figure 4B), or between MRLlprTLR4−/− mice and littermate controls (figure 4C). We also analysed levels of IgG subclasses and IgM at 18 weeks in all mice. There were no signficant differences when MRLlpr mice and TLR4 deficient MRLlpr littermate controls as shown in figure 5 A–J. When MRLlpr mice and TLR2 deficient MRLlpr littermate controls were compared there were higher levels of IgG2a and IgG2b, but no difference in levels of other subclasses or IgM. Levels of anti-ssDNA antibodies were also measured with no significant differences found (figure 5 K–L).

## Discussion

In this study we have demonstrated that neither TLR2 nor TLR4 deficiency modulates autoantibody production or glomerulonephritis in MRLlpr mice. These data suggest that in the presence of a strong genetic predisposition to lupus, the role of TLR2 or TLR4 is not significant. Although the result is surprising given the previous data in milder lupus models suggesting these TLRs are important, our data do not in any way invalidate or question the results obtained in these previous studies. In our study, care was taken to use littermate controls and to confirm homozygosity for the MRL H^2^ haplotype, meaning that genetic variation is very unlikely to account for the lack of an observed difference between the cohorts. Robust disease was seen, with some mortality, significant proteinuria, and severe glomerulonephritis at 18 weeks which is consistent with published reports.

Our data cannot exclude the possibility that there were histological differences between groups at timepoints earlier than 18 weeks. However there were no differences in the survival curves, and when we analysed anti-dsDNA antibodies at week 14 and there were no differences. A difference in the tempo of disease induction therefore seems unlikely. We did find higher levels of IgG2a and IgG2b in TLR2 deficient MRLlpr mice. The significance of this is not clear, but it does not suggest that TLR2 deficiency inhibits the autoimmune phenotype.

Proposed endogenous ligands for TLR2 and TLR4 include heat-shock proteins, heparan sulphate, fibrinogen, biglycan, hyaluronic acid and high mobility group box protein 1(HMGB1). These molecules are likely to be released during end-organ inflammation such as glomerulonephritis in SLE. These ligands have been shown to activate TLR2 and TLR4 in both in vitro and also in vivo systems (13–18). Therefore, even in the absence of an effect on autoantibody levels, we may have anticipated an effect on the severity of glomerulonephritis. A recent report has demonstrated a role for biglycan in MRLlpr lupus [18]. Overexpression exacerbated disease, and deficiency diminished disease. The mechanism of this effect was thought to depend on the B cell chemokine CXCL13 which was released in a TLR2/TLR4-dependant manner. This finding would predict that lupus would be attenuated in MRLlpr mice deficient in TLR2 or TLR4. There are at least two potential reasons for this discrepancy. Firstly biglycan is also a ligand for the receptor PTX7 although the release of CXCL13 was found to depend on TLR2/4 rather than PTX7. Nonetheless, there may be an additional role for PTX7 in biglycan-mediated effects. In addition, there may be some redundancy since biglycan may act via either TLR2 or TLR4. Therefore we cannot be sure that combined deficiency of TLR2 and TLR4 would not have resulted in protection from lupus in MRLlpr mice where none was seen with deficiency in just one receptor. HMGB1, a nucleosome binding protein has been shown to be present at increased levels in the plasma of SLE patients, and to stimulate dsDNA antibody production in mice in TLR dependent manner [19]. HMGB1 has also been shown to act via both TLR2 and TLR4 which further raises the possibility that deficiency a combined deficiency of both TLR2 and TLR4 might have shown an effect MRLlpr mice. These experiments would certainly be of interest and could form the basis for future work.

## Conclusions

Our data directly examines the role of TLR2 and TLR4 in one of the commonly used strains with a genetic predisposition to robust lupus-like disease. We have not demonstrated a role for TLR2 or TLR4, and we suggest that these may not be useful therapeutic targets in SLE. Further preclinical data in other lupus-prone strains will be required to clarify this further.
